# Oral semaglutide for the treatment of obesity: a retrospective real-world study

**DOI:** 10.3389/fendo.2025.1593334

**Published:** 2025-05-29

**Authors:** Mitja Krajnc, Neža Kuhar, Andrijana Koceva

**Affiliations:** ^1^ Department of Endocrinology and Diabetology, Clinic of Internal Medicine, University Medical Centre Maribor, Maribor, Slovenia; ^2^ Faculty of Medicine, University of Maribor, Maribor, Slovenia

**Keywords:** obesity, oral semaglutide, GLP-1 receptor agonist, efficacy, safety, real-world evidence, weight loss

## Abstract

**Background:**

Clinical obesity is a prevalent chronic disease, significantly increasing morbidity and mortality while impairing quality of life. As diet and physical activity interventions often prove ineffective in the long term, with increasing use of pharmacotherapy, drug shortages and injection aversion present a challenge. The role of oral semaglutide at a dose of 14 mg (registered for type 2 diabetes) as a treatment for obesity in patients without diabetes remains undefined.

**Methods:**

In the retrospective real-world study, which included 93 adults without diabetes (57% women, average age 52 years), we assessed whether treatment with 14 mg oral semaglutide over one year is associated with lower body weight, body mass index (BMI), waist circumference, blood pressure, heart rate, and obesity staging according to EOSS.

**Results:**

Of the 93 subjects recruited, 82 (88%) were receiving oral semaglutide at a dose of 14 mg after one year. After one year of treatment body weight was significantly lower by 5.7% (5.9 kg) in completers, and BMI decreased significantly by an average of 2 kg/m². There was also a significant reduction in waist circumference by 5.5 cm and a decrease in EOSS score by 0.1. Clinically significant weight loss was achieved in 46% of all participants, with rare individuals experiencing a decrease of ≥ 15%. Adverse effects were mostly mild, with nausea reported by 23% and vomiting and diarrhoea by 12% of participants.

**Conclusions:**

Obesity treatment with oral semaglutide at a dose of 14 mg showed marked interindividual variability, with approximately half achieving clinically significant reductions – mostly under 10%. Although less effective than injectable therapy, oral semaglutide at a dose of 14 mg had a favorable safety profile and may be suitable in selected clinical scenarios.

## 1 Introduction

Globally, 13% of adults are obese, while in Slovenia, based on data from 2020, approximately 20% of adults aged 25 to 74 are obese, and 39% are overweight (1, 2). Obesity is significantly associated with adverse health outcomes, quality of life and increased mortality. Clinically, its definition is based on the body mass index (BMI), which is calculated as the ratio of body weight to the square of height. In Caucasian individuals, a BMI of 30 kg/m² or higher is considered obese, although the known limitations of BMI must be acknowledged (1, 3). Abdominal fat distribution, e.g. measured by waist circumference, is independently associated with cardiovascular disease and mortality, regardless of BMI (4).

In obesity management, apart from assessing nutritional status and physical capacity, it is crucial to identify individual causes and contributing factors and evaluate medical, functional, and psychological complications (5, 6). The Edmonton Obesity Staging System (EOSS) is commonly used to assess obesity severity. Higher EOSS categories are independently associated with greater overall and specific mortality in individuals with obesity (7). The primary goal of obesity treatment is to improve health, with weight monitoring serving as a proxy indicator of treatment efficacy. A weight loss of at least 5% is considered clinically significant, while a 15% reduction effectively improves most obesity-related complications and conditions. The target weight loss varies depending on the specific complications associated with obesity (1, 6, 8).

The foundation of obesity treatment is lifestyle modification, the long-term effectivness of which is often limited and insufficient, even in intensive lifestyle programs. Pharmacological intervention is indicated for individuals with obesity (BMI > 30) or overweight (BMI > 27) if comorbidities are present (9–11). Semaglutide is a synthetic GLP-1 receptor agonist (GLP-1 RA). It mimics the action of the natural hormone but has improved stability and a longer half-life, allowing for less frequent dosing (12). Semaglutide is the active ingredient in Ozempic^®^ and Rybelsus^®^, which are registered for type 2 diabetes treatment and Wegovy^®^, which is indicated for obesity treatment. Wegovy pens contain higher doses of semaglutide (up to 2.4 mg weekly), whereas the maximum Ozempic dose is 1 mg weekly (13, 14). Beyond their beneficial effects on blood glucose and weight, GLP-1 RAs are associated with a reduced risk of numerous conditions, including cardiovascular events and a reduction in mortality (16). Oral semaglutide (Rybelsus^®^) is currently approved for the treatment of adults with type 2 diabetes who have not achieved adequate glycemic control with non-pharmacological measures. It is available in three daily doses (3 mg, 7 mg, and 14 mg), with dose escalation every four or more weeks (15). The efficacy and safety of oral semaglutide (Rybelsus) for type 2 diabetes have been extensively studied in the PIONEER trials (17) and a 14 mg daily dose of Rybelsus is considered equivalent to 0.5 mg Ozempic weekly, although oral absorption variability is significantly higher (18). Favorable results on weight have been reported for higher doses of oral semaglutide (25 and 50 mg daily), which are not yet in clinical use (19). And injectable semaglutide has demonstrated significant weight loss efficacy with mild to moderate gastrointestinal-related side effects and treatment discontinuation in only 7% of participants (20).

In Slovenia, access to effective obesity medications remains limited and pharmacological obesity treatment is not reimbursed. Wegovy^®^ is unavailable, Ozempic^®^ is difficult to obtain due to global shortages (21), and tirzepatide is newly available but at a higher price. Many patients proactively request semaglutide tablets for weight loss. A review of existing literature using keywords such as oral semaglutide, obesity and weight loss revealed a lack of reliable data on the use and efficacy of oral semaglutide, at doses approved for type 2 diabetes, in individuals with obesity but without diabetes. In response, we retrospectively evaluated the efficacy and safety of oral semaglutide at a dose of 14 mg for obesity treatment in this population.

## 2 Materials and methods

### 2.1 Study design and population

Our study was retrospective and observational, based on real-world data from clinical practice. We reviewed and analyzed available medical records (both electronic and paper-based reports) of adults (aged ≥ 18 years) who, by current professional guidelines for obesity treatment (8, 9, 11), were prescribed oral semaglutide (Rybelsus) between 2021 and 2023 for obesity treatment (defined as a BMI ≥30 kg/m²). The prescribed treatment followed the dosing regimen for type 2 diabetes: 3 mg on an empty stomach for the first 30 days, 7 mg on an empty stomach for the next 30 days (second month), and 14 mg on an empty stomach daily from the third month onward. Participants were managed in one of two specialized endocrinology outpatient clinics (either in a hospital or a private center) where obesity management is routinely conducted.

We excluded individuals with concomitant diabetes of any type, those who were overweight or had normal body weight, and individuals with confirmed or suspected syndromic or monogenic obesity. We also excluded individuals whose BMI was likely attributable to increased muscle mass rather than excess body fat, as BMI may overestimate obesity in such cases. The contraindications and precautions stated in the approved Summary of Product Characteristics were considered during prescription (18). Women of childbearing potential were advised to use a reliable method of contraception during the treatment period.

After reviewing the medical records of all patients prescribed oral semaglutide for obesity in both outpatient clinics, we included those who provided informed consent for data collection and analysis in the study. Participants were eligible if we had the following data available at the start of treatment (day 0) and at the follow-up after one year (365 ± 14 days):

- Before treatment initiation: sex, age (years), body weight (kg), waist circumference (cm), height (cm), body mass index (BMI), systolic and diastolic blood pressure, heart rate, and Edmonton Obesity Staging System (EOSS) score.
- After one year for those taking Rybelsus 14 mg daily: age (years), body weight (kg), waist circumference (cm), height (cm), BMI, systolic and diastolic blood pressure, heart rate, adverse effects related to the medication (type, severity), and EOSS score.
- After one year for those who discontinued or did not reach the 14 mg dose: adverse effects related to the medication (type, severity) and reason for treatment discontinuation.

The study included 93 individuals. During the first outpatient visit, participants received a brief (approximately 30-minute) focused counselling on non-pharmacological treatment of obesity, based on national guidelines (11). This included dietary advice and recommendations regarding physical activity. All patients were also encouraged to enroll in a free structured, multidisciplinary lifestyle modification weight loss program at a local health center. The program, consisting of 30 sessions over 4 months, focuses on acquiring practical knowledge and skills to change dietary and physical activity habits and provides cognitive-behavioural psychological support.

Body weight measurements were performed using a calibrated personal scale, verified by an accredited laboratory. Participants were advised to fast before weighing. Body weight results were rounded to the nearest kilogram. Height was measured in a standing position from the crown of the head to the soles of the feet and rounded to the nearest centimeter. BMI was calculated using the standard formula. Waist circumference was measured following WHO recommendations, at the midpoint between the lowest palpable rib and the highest point of the iliac crest, rounded to the nearest centimeter. Systolic and diastolic blood pressure and heart rate were measured using a calibrated automatic upper-arm blood pressure monitor employing the oscillometric method, following the guidelines of the European Society of Cardiology (22). The EOSS obesity staging score was assessed by an endocrinologist based on published criteria (23).

Due to the retrospective nature of the study, no medical interventions were performed on participants as part of the research, all data used in the study had been collected during routine outpatient care. The medication had been prescribed by endocrinologist involved in obesity treatment, with the participants’ consent and agreement. Although semaglutide has well-documented clinical benefits, participants were informed that oral semaglutide, unlike the injectable form, is not officially approved for obesity treatment. All participants provided informed consent for data collection and analysis in an anonymized format, obtained by their treating specialist. Our institution’s Medical Ethics Committee ethically reviewed and approved the study.

### 2.2 Statistical analysis

We conducted a power analysis for the study, considering a minimally relevant effect size (δ) of a 5 kg weight change, a two-tailed test, a minimum desired power of 0.9, and α = 0.05. Using a paired t-test, we determined that the sample size in our study was statistically sufficient.

The Shapiro-Wilk test was used to assess the normality of variable distributions and the distribution of differences in values before and after treatment. Normally distributed variables were described using the mean and standard deviation, while non-normally distributed variables were reported using the median and interquartile range.

Relative changes in body weight were calculated using the formula: $\Delta BW(\%)=\frac{BW_{after}-BW_{before}}{BW_{before}}$

We categorized participants based on their weight loss percentages (≥5%, 10%, and 15%) and reported the proportions of individuals experiencing specific adverse effects at mild, moderate, or severe levels. To compare pre- and post-treatment data, we used the paired Student’s t-test when the assumption of normality was met. Otherwise, we applied the Wilcoxon signed-rank test. We also calculated 95% confidence intervals. To compare the subgroups of completers and non-completers, we used the unpaired sample Student’s t-test or the Mann-Whitney U test, respectively.

Statistical analysis was conducted using the latest open-source software, Jamovi (2.6.2), available online at https://www.jamovi.org. A p-value < 0.05 was considered statistically significant.

## 3 Results

After one year, 82 subjects (88%) were receiving oral semaglutide at a dose of 14 mg (completers), while 11 subjects or 12% (non-completers) had either discontinued treatment earlier due to adverse effects or lack of efficacy, or were taking a lower dose (7 mg daily). Most completers (49%) were initially classified as EOSS stage 2, 28% as stage 1, 17% as stage 3, and one participant as stage 4, the rest as stage 0. Only three participants joined a structured, multidisciplinary lifestyle intervention program, all of which within the first two months of the initial visit.

We compared the baseline characteristics of the subgroups of completers and non-completers. No statistically significant differences were found between the subgroups for any of the characteristics (p>0.05 for all comparisons). **Table 1** presents the baseline demographic and clinical characteristics of all participants, while **Table 2** presents the differences in assessed parameters and their significance between the baseline and the one-year follow-up in 82 completers.

**Table 1:** Demographic and clinical characteristics of study participants before initiating treatment.

Variable	Completers, n=82	Non-completers, n=11
Age	51.3 ± 12.5	53.8 ± 15.0
Female sex – number (%)	47 (57%)	6 (55%)
Height (cm)	171±9	168±11
Body weight (kg)	105 ± 18	107 ± 12
Waist circumference (cm)	113±13	111±12
Systolic blood pressure (mmHg)	137±15	140±13
Diastolic blood pressure (mmHg)	86 (12)*	88 (11)*
Heart Rate (beats/min)	79±11	81±15
EOSS score	2 (1)*	2 (0.5)*
BMI	35 (6)*	36 (6)*
Caucasian – number (%)	82 (100)	11 (100)

Data for the 82 completers are presented in the left column, and data for the 11 non-completers in the right column.

Except for sex, values are given as mean ± SD, and for non-normally distributed variables (marked with *), as median (interquartile range).

**Table 2:** Changes in assessed parameters between the one-year follow-up and baseline and their statistical significance in completers (n=82).

Change in the assessed parameter	Value	95% Confidence Interval	p-value
Relative body weight (%)	-5.7	-6.7,-4.6	
Absolute body weight (kg)	-5.9	-7.0,-4.9	<0.001
Waist circumference (cm)	-5.5	-6.6,-4.7	<0.001
Systolic blood pressure (mmHg)*	-1.0	-3,1	0.27
Diastolic blood pressure (mmHg)*	-1.0	-2,0	0.21
Heart rate (beats/min)*	1	0,1.5	0.005
EOSS score*	-0.1	-0.11/-0.02	0.01
BMI	-2.0	-2.4/-1.7	<0,001

The table includes p-values for the paired Student’s t-test for normally distributed differences and for the Wilcoxon test (marked with *) for non-normally distributed data. The lower and upper bounds of the 95% confidence interval are provided. Values of p<0.05 are statistically significant.

**Figure 1:**
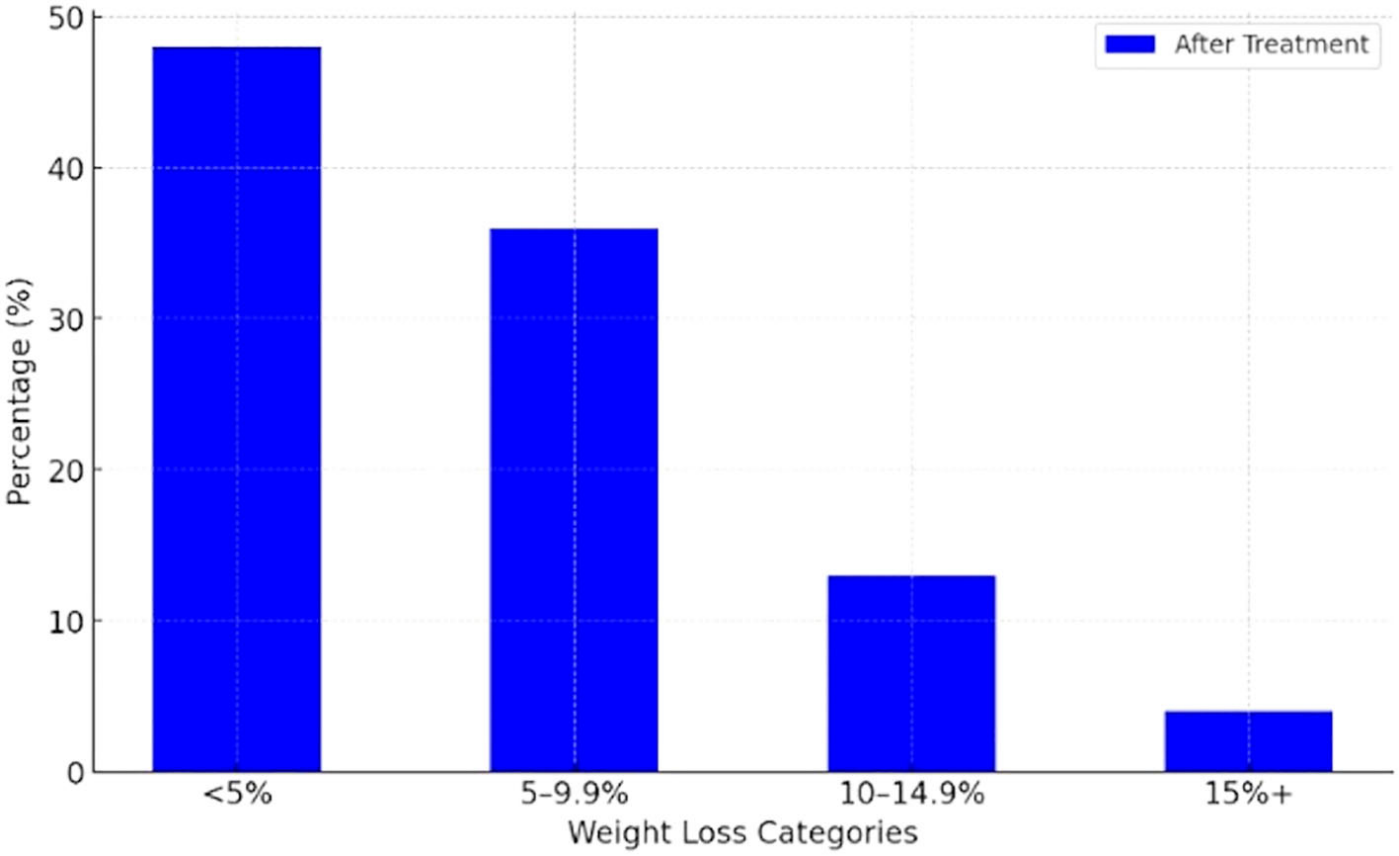
Distribution of completers by weight loss categories (n=82).

**Figure 2:**
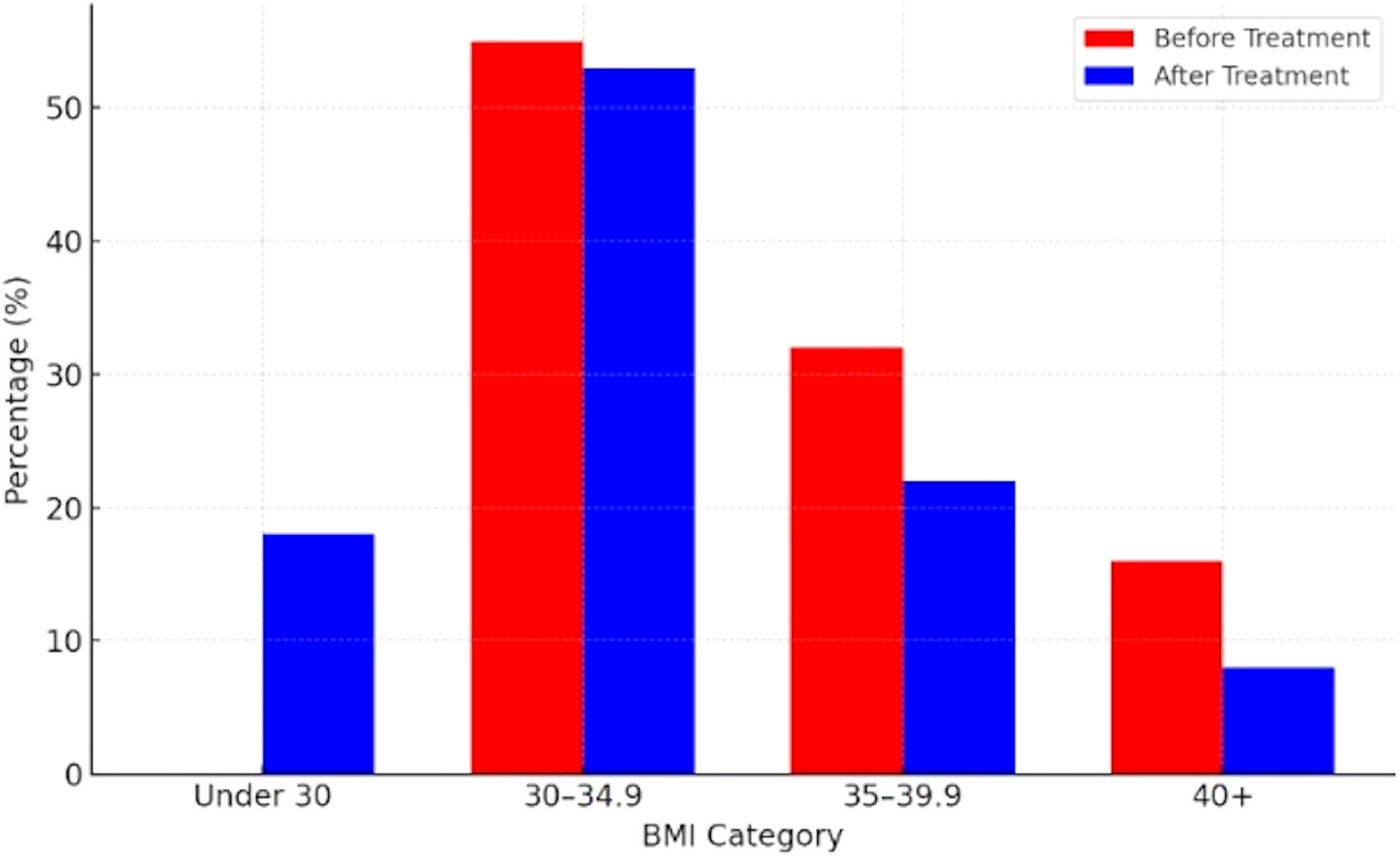
Proportion of completers by BMI category before and after treatment (n=82).

The maximal relative weight change in completers was -17%, and the minimal was +3%. After one year, 48% had lost less than 5% of their body weight, while 4% had lost more than 15% of their body weight. Data on distribution of completers by their weight loss categories are presented in **Figure 1**, while **Figure 2** presents the proportion of completers by BMI categories at the baseline and after one year of treatment.

We also observed a significant decrease in waist circumference that was less than with tirzepatide at all maintenance doses, subcutaneous semaglutide 2.4 mg weekly, and oral semaglutide at a dose of 50 mg daily (19, 20, 27). There was also a mild decrease in systolic and diastolic blood pressure, but these changes were not statistically significant. A small but significant increase in the heart rate (by 1 bpm) was noted, which, according to the literature, has no meaningful health implications.

Of the 93 participants in our study, 11 patients (12%) discontinued treatment. Five non-completers discontinued treatment due to self-perceived insufficient weight loss, while six could not take the recommended dose due to adverse effects. Among these six, two experienced severe vomiting at the dose of 14 mg, three had moderate to severe nausea, and one had severe diarrhoea. Of these six, two were taking a lower dose (7 mg) and tolerated it well. The most frequently reported side effect among the completers in our study was nausea, affecting 23% of participants. Vomiting and diarrhoea were the next most common, each affecting 12%. Constipation and nonspecific abdominal pain followed, each reported by 6% of participants. None of the participants experienced hypoglycemia, newly developed or worsened depressive disorders, or newly diagnosed gallbladder diseases. None of the participants experienced a serious adverse event as defined by the European Medicines Agency (EMA) (24), and no confirmed case of acute pancreatitis was found. **Table 3** presents reported adverse events that completers attributed to the medication after one year of treatment with oral semaglutide 14 mg daily.

**Table 3:** Adverse events reported by completers after one year of treatment.

Adverse event	n (%)
Nausea	19 (23)
- mild	13 (16)
- moderate	6 (7)
- severe	0
Vomiting	10 (12)
- mild	8 (10)
- moderate	2 (2)
- severe	0
Diarrhoea	10 (12)
- mild	10 (12)
- moderate	0
- severe	0
Constipation	5 (6)
- mild	5 (6)
- moderate	0
- severe	0
Abdominal pain	5 (6)
- mild	3 (4)
- moderate	2 (2)
- severe	0
Hypoglycemia	0
Depression (new or worsening)	0
Gallbladder inflammation or gallstones	0
Acute cardiovascular event	0
Serious adverse event (EMA)	0
Death	0

Serious adverse events were defined according to the European Medicines Agency (EMA) criteria (24).

## 3 Discussion

Eighty-two participants, who continued with 14 mg oral semaglutide (the highest dose registered for managing type 2 diabetes) for one year experienced a significant average weight loss of 5.7% (5.9 kg), along with a reduction in BMI by an average of 2 kg/m². While the average weight loss reached the clinically significant threshold of ≥ 5%, a notable 48% of completers did not meet this criterion. Overall, 54% of all 93 enrolled individuals failed to achieve the 5% weight loss target. Only 4% of completers experienced a weight reduction of 15% or more, which is associated with substantial improvements in various obesity-related negative outcomes (8). Our findings indicate a considerable interindividual variability and relatively modest effect of oral semaglutide ar the studied dose on body weight, which is predominantly mild to moderate.

There are some head-to-head comparative studies between anti-obesity drugs, e.g. for subcutaneous semaglutide and tirzepatide in a large cohort study or subcutaneous semaglutide and liraglutide in a randomized trial (25, 26). For oral semaglutide in adults without diabetes, the following comparisons are based on studies investigating individual drugs, and differences in participant characteristics, study designs, and treatment durations should be noted.

According to our findings and published data, oral semaglutide at a dose of 14 mg is significantly less effective for weight loss in individuals with obesity than tirzepatide (a dual GLP-1 and GIP agonist). In a randomized controlled trial, tirzepatide at doses of 10 mg and 15 mg resulted in an average weight reduction of 20% and 21%, respectively, while the 5 mg dose led to a 15% weight loss, compared to 3% with placebo. Clinically significant weight loss of at least 5% was achieved by 89% and 91% of participants, while 67–71% lost at least 15% of their body weight. Among our participants, nausea incidence was comparable to the lowest maintenance dose of tirzepatide, while fewer reported constipation or diarrhoea (27). Real-world data also indicate that most users of tirzepatide without diabetes lost more than 10% of body weight with an average loss of 12.7% (28).

In a randomized controlled trial invastigating the efficacy of subcutaneous semaglutide injections at a dose of 2.4 mg weekly for obesity treatment, participants experienced an average weight loss of 15% and a BMI reduction by an average of 5.5 kg/m² after 68 weeks (20). More than 86% of participants achieved clinically significant weight loss of at least 5%, and 51% of participants lost over 15% of their body weight (20). Compared to our study, a slightly lower percentage of participants reported nausea (17%), diarrhoea (16%), and vomiting (7%), which is unexpected given that their dose and exposure to semaglutide were significantly higher. However, 6% of participants reported serious adverse events, and 3% discontinued treatment due to side effects. Unlike our study, some cases of hypoglycemia and gallbladder disease were reported (20). In a large real-world study, only 10% of users followed recommended dose increases for subcutaneous semaglutide but they generally resembled trial participants (29). Weight loss in a regular clinical setting was similar to weight loss in randomized clinical trials (30).

In the PIONEER 1 study (31), which investigated monotherapy with oral semaglutide in type 2 diabetes, participants on 14 mg semaglutide experienced an average weight loss of 4 kg after 26 weeks (31). At least 5% weight loss was achieved by 41% of participants (compared to 15% with placebo), which is similar to our results. However, BMI in PIONEER 1 was 32 on average (31), lower than in our study, and semaglutide is known to be less effective for weight loss in individuals with type 2 diabetes. In our study, nausea, vomiting, or constipation were reported at slightly higher rates. Given that participants in real-world conditions were aware of their treatment and could be well-informed about potential side effects, a nocebo effect might have contributed to these results.

Additionally, in the OASIS 1 trial (19), oral semaglutide at a dose of 50 mg, which is not yet available for clinical use in obesity or type 2 diabetes, induced an average of 15% weight loss, with 85% of participants achieving at least 5% weight loss (compared to 26% with placebo) and 54% achieving at least 15% weight loss. Participants BMI also decreased by an average of 5.6 kg/m² (19).

According to our data and published data on other medications, we can indirectly infer that oral semaglutide at a dose of 14 mg is associated with less weight loss and a smaller reduction in BMI compared to tirzepatide at all maintenance doses, subcutaneous semaglutide at 2.4 mg weekly, and oral semaglutide at 50 mg daily. The weight loss effect of 14 mg oral semaglutide appears numerically comparable to older obesity treatments: orlistat (360 mg daily) with 8% weight loss and 51% achieving at least 5% weight loss, naltrexone-bupropion (32/360 mg) with 6% weight loss and with 48% achieving at least 5% weight loss and liraglutide (3 mg daily) with 8 kg weight loss and 63% achieving at least 5% weight loss (1, 9). The most common adverse effects of oral semaglutide are gastrointestinal, including nausea, diarrhea, vomiting, constipation, dyspepsia and upper abdominal pain. Most adverse effects are mild to moderate, transient, and not associated with treatment discontinuation. During treatment, monitoring is required regarding the potential occurrence of acute pancreatitis, gallbladder disease, suicidal ideation, medullary thyroid carcinoma, acute kidney injury, and worsening of diabetic retinopathy (15, 17, 18). In our study the most frequently reported side effect was nausea, affecting 23% of participants, followed by vomiting and diarrhoea, each affecting 12%, and constipation and nonspecific abdominal pain reported by 6% of participants. The majority of adverse events were mild, and although among our participants 6% discontinued treatment due to side effects, none experienced a serious adverse event.

The retrospective and observational design of our study facilitated faster execution, however, several limitations must be acknowledged. The risk of selection bias is inherent to retrospective observational studies. In our study, pharmacological treatment with oral semaglutide was prescribed only to patients who were both able to self-fund their treatment and motivated to seek obesity care. Due to the non-randomized and observational design of the study, causality cannot be established. Nonetheless, the magnitude of weight loss observed exceeded what would be anticipated from placebo or lifestyle modifications alone, particularly in the absence of a structured diet and physical activity program, which only three participants attended. This likely minimized the potential confounding influence of intesive lifestyle modification. Additionally, the low number of lifestyle program participants precluded any meaningful comparison between participants and non-participants, and the overall limited sample size restricted the ability to conduct subgroup analyses in general. Furthermore, the absence of a comparator group also weakens the robustness of the conclusions. Finally, the study was conducted in Caucasian adults, limiting the findings’ generalizability to more diverse populations.

From a clinical perspective, oral semaglutide may be particularly useful in patients who decline injectable therapy, when injectable medications are unavailable, or as an initial therapy before transitioning to more potent treatment options. Considering the ongoing shortage of injectable incretin-based therapies, future research should aim to prioritize randomized controlled trials evaluating the efficacy of oral semaglutide in patients without diabetes, as well as include a broader demographic spectrum, integrate pharmacological treatment with structured non-pharmacological interventions and improve control over potential confounders, especially those related to lifestyle.

## 5 Conclusions

In conclusion, the present study, which evaluated oral semaglutide at a daily dose of 14 mg for the treatment of obesity in adults without diabetes, demonstrated substantial interindividual variability in weight loss response in a real-world setting. Approximately half of the participants experienced a clinically significant reduction in body weight, although this reduction was mostly less than 10%. The treatment demonstrated a favorable safety profile, with no major safety concerns observed.

## Data Availability

The raw data supporting the conclusions of this article will be made available by the authors, without undue reservation.
